# Epigenetic mechanisms of Müller glial reprogramming mediating retinal regeneration

**DOI:** 10.3389/fcell.2023.1157893

**Published:** 2023-06-15

**Authors:** Tian-En Si, Zhixiao Li, Jingjing Zhang, Songxue Su, Yupeng Liu, Shiyue Chen, Guang-Hua Peng, Jing Cao, Weidong Zang

**Affiliations:** ^1^ Department of Anatomy, Basic Medical College, Zhengzhou University, Zhengzhou, China; ^2^ Department of Pathophysiology, Basic Medical College, Zhengzhou University, Zhengzhou, China; ^3^ Laboratory of Visual Cell Differentiation and Regulation, Basic Medical College, Zhengzhou University, Zhengzhou, China

**Keywords:** Müller glia reprogramming, retinal regeneration, epigenetics, histone, DNA methylation, miRNA

## Abstract

Retinal degenerative diseases, characterized by retinal neuronal death and severe vision loss, affect millions of people worldwide. One of the most promising treatment methods for retinal degenerative diseases is to reprogram non-neuronal cells into stem or progenitor cells, which then have the potential to re-differentiate to replace the dead neurons, thereby promoting retinal regeneration. Müller glia are the major glial cell type and play an important regulatory role in retinal metabolism and retinal cell regeneration. Müller glia can serve as a source of neurogenic progenitor cells in organisms with the ability to regenerate the nervous system. Current evidence points toward the reprogramming process of Müller glia, involving changes in the expression of pluripotent factors and other key signaling molecules that may be regulated by epigenetic mechanisms. This review summarizes recent knowledge of epigenetic modifications involved in the reprogramming process of Müller glia and the subsequent changes to gene expression and the outcomes. In living organisms, epigenetic mechanisms mainly include DNA methylation, histone modification, and microRNA–mediated miRNA degradation, all of which play a crucial role in the reprogramming process of Müller glia. The information presented in this review will improve the understanding of the mechanisms underlying the Müller glial reprogramming process and provide a research basis for the development of Müller glial reprogramming therapy for retinal degenerative diseases.

## 1 Introduction

Loss of photoreceptors in retinal degenerative diseases (such as retinitis pigmentosa) and age-related macular degeneration can lead to irreversible loss of vision. Furthermore, effective clinical treatment is still lacking for retinal degenerative diseases, thus demanding the development of more effective treatment and prevention methods (Age-Related Eye Disease Study Research, 2001; Chew et al., 2014; Wahlin et al., 2017). Anti-inflammatory and neurotrophic methods are often used in the clinical treatment of retinal degenerative diseases, but their effect is very limited and cannot effectively restore the function of damaged retinal pigment epithelial (RPE) cells, photoreceptor cells, and other retinal neurons (Seddon et al., 1994; Chew et al., 2013). Transplantation of exogenous stem cells or activation of endogenous precursor cells can promote the regeneration of retinal neurons, thus alleviating the severity of retinal degenerative diseases (Koso et al., 2009; Luo et al., 2014; Santos-Ferreira et al., 2016). Exogenous cell sources include retinal organoids and cells derived from the differentiation of induced pluripotent stem cells (such as RPE cells). However, retinal cell replacement therapy has major risks and challenges owing to immune rejection and other problems (Singh et al., 2013a; da Cruz et al., 2018). Therefore, increasing interest has been paid to cell reprogramming techniques, which promote retinal regeneration by reprogramming non-neuronal cells in the body into stem cells, thereby activating potential repair pathways in damaged tissues (e.g., activating endogenous stem cells) (Yao et al., 2018). Cell reprogramming technology is currently being applied to regenerate various cell types in the pancreas, heart, liver, and central nervous system and is expected to emerge as a new method for the treatment of retinal degenerative diseases (Singh et al., 2013b; Mandai et al., 2017; Waldron et al., 2018; Yao et al., 2018).

Müller glia are the main type of glial cell in the vertebrate retina and are widely associated anatomically and functionally with cell bodies and protrusions of neurons in all layers of the retina. Müller glia are one of the last retinal cell types that differentiate during development, and their main function is to maintain retinal homeostasis and integrity (Fischer et al., 2009; Langmann et al., 2010). Müller glia are involved in neuronal excitatory regulation and glucose metabolism (Newman, 2015; Ma et al., 2019). Moreover, Müller glia are involved in retinal regeneration through reprogramming, a process that involves the dedifferentiation of Müller glia, their migration to the injury site, and redifferentiation into new retinal cells to repair the damaged area (Ueki et al., 2015; Jorstad et al., 2017). In zebrafish and other lower vertebrate models, retinal Müller glial cells dedifferentiated into retinal precursor cells and further differentiated into mature photoreceptors, ganglion cells, and interneurons (Thummel et al., 2008; Lenkowski et al., 2013). However, compared with lower vertebrates, the differentiation probability of mammalian Müller glial cells under degenerative or injured conditions is very low, which does not meet the requirements for retinal self-healing (Ahmad et al., 2020; Bonilla-Pons et al., 2022). Recently, based on the understanding of the reprogramming mechanism of Müller glial cells in zebrafish, appropriate tools and methods have been established to improve the induction of Müller glial reprogramming in mammals. In mature Müller glia, the loss of reprogramming ability is accompanied by reduced chromatin accessibility, suggesting the implication of epigenetic factors in retinal regeneration (Jorstad et al., 2017). Epigenetics mainly refers to the process of stem cell differentiation as part of the eukaryotic cell differentiation process. Reprogramming of Müller glia involves epigenetic alterations, but the specific mechanism remains unclear (Huangfu et al., 2008; Gao et al., 2013; Lu et al., 2020). Retinal damage alters the DNA methylation status of Müller glia and initiates the differential expression of Müller glia histone-modifying enzymes (Powell et al., 2013; Ueno et al., 2017). Moreover, transcription factors such as Atoh7 and ASCL1 can induce the reprogramming of Müller glial cells into retinal progenitor cells (RPCs) in the absence of retinal injury (Todd et al., 2021). Multipotent transcription factors including ASCL1, SOX2, OCT4, and MYC play an important role in the reprogramming of zebrafish Müller glia (Qin et al., 2009; Todd et al., 2022). In a study of a mouse model of retinal injury, histone deacetylase inhibitors promoted accessibility at key gene loci ASCL1 in the Müller glia and induced more effective reprogramming (Jorstad et al., 2017). These findings indicate that Müller glia can be dedifferentiated into retinal precursor cells by epigenetically regulating the expression of certain transcription factors, thereby regulating the retinal regeneration process; this approach opens up new possibilities for the treatment of retinal degenerative diseases. Therefore, we conducted a review of the current understanding of epigenetic regulation of Müller glial reprogramming and its potential mechanism, with particular emphasis on the therapeutic potential of Müller glial cells in retinal degenerative diseases; these findings can serve as a reference for future research on regenerative therapy for retinal degenerative diseases.

## 2 DNA methylation modifications in Müller glial reprogramming

DNA methylation is a chemical modification of the DNA that can alter genetic expression without altering the DNA sequence (Wu and Zhang, 2014). DNA methylation is a process wherein a methyl group binds to the covalent bond at the carbon 5 position of the cytosine ring of a genomic CpG dinucleotide, catalyzed by DNA methyltransferases (Wu and Zhang, 2014). DNA methyltransferases mainly include DNMT1, DNMT3a, DNMT3b, etc. DNMT1 is responsible for maintaining DNA methylation patterns during DNA replication, while members of the DNMT3 enzyme family have their primary role in *de novo* DNA methylation (Chen et al., 2002; Laget et al., 2014). Moreover, DNMT3a and DNMT3b are highly expressed in undifferentiated embryonic stem (ES) cells. To this end, studies have shown that DNA methylation can cause changes in chromatin structure, DNA conformation, DNA stability, and the way DNA interacts with proteins, thereby regulating gene expression (Jones, 2012). Methylated DNA can undergo demethylation, catalyzed by DNA demethylase enzymes. Among the demethylase enzymes, 5-methylcytosine DNA glycosylase is predominantly found in organisms. Additionally, the TET demethylase family and methylated CpG-binding proteins such as MBD2 also possess demethylase activity (Onodera et al., 2021). DNA demethylation is a process wherein the methylated bases are removed in the presence of DNA glycosidases, which is equivalent to the repair reaction of damaged DNA catalyzed by glycosidase and base-free nuclease digestion coupling. In line with this, some studies suggest that DNA demethylation is important to turn on the expression of specific genes (Zhao et al., 2008; Zeng and Chen, 2019).

Furthermore, 100% of RPC-associated gene promoters and 99% of cell cycle–associated gene promoters were located in unmethylated or hypomethylated regions of the Müller glial cell genome, suggesting that Müller glial cells are epigenetically similar to RPC-like phenotypes (Dvoriantchikova et al., 2019). Nevertheless, many specific pluripotent transcription factors necessary for the regeneration of retinal neurons are not expressed in Müller glial cells, such as Atoh7, ASCL1, and Neurog2 (Bernardos et al., 2007; Ramachandran et al., 2015). Combinations of different transcription factors including ASCL1, Pou4f2, Islet1, and Atoh1 are expressed in genetically engineered mouse model, and these factors promote retinal cell regeneration (Todd et al., 2022). Moreover, the activation of pluripotency factor transcription gene in Müller glia is closely related to DNA demethylation. Thus, this finding indicates that DNA methylation might be involved in the reprogramming of Müller glial cells after retinal injury (Powell et al., 2013).

Luis et al. investigated the expression of early pluripotency-related genes such as OCT4, ASCL1, and LIN28 and their methylation alterations in an experimental mouse model of retinal damage; these genes were reported to be essential for retinal regeneration (Gao et al., 2013; Reyes-Aguirre and Lamas, 2016). Notably, peak expression of the pluripotent gene OCT4 was observed at the early stage of injury and was associated with a significant decrease in the expression level of the methylation transferase DNMT3b (Deng et al., 2021). Given the apparent correlation between Oct4 and DNMT3B, Müller glial dedifferentiation after mammalian retinal injury may be limited by DNA methylation. Methylation rapidly silenced Oct4 expression and prevented the acquisition of pluripotency. The use of the DNA methyltransferase inhibitor SGI-1027 could reverse this effect, suggesting that a decrease in the DNA methylation of pluripotency genes induces the dedifferentiation process in Müller glia.

ASCL1 is essential for the dedifferentiation and regeneration processes of zebrafish Müller glia during retinal regeneration and reprogramming of mouse Müller glia into retinal neurons *in vitro* and *in vivo* (Pollak et al., 2013; Ueki et al., 2015; Gorsuch et al., 2017; Elsaeidi et al., 2018). Notably, ASCL1 is a pluripotent transcription factor essential for retinal regeneration in zebrafish. To this end, ASCL1 and its downstream target, namely, Lin28a/b, are highly expressed in RPCs but are absent in Müller glial cells (Elsaeidi et al., 2018). However, ASCL1 expression is rapidly upregulated in Müller glial cells after retinal injury (Ramachandran et al., 2015; Ueki et al., 2015). Likewise, DNA demethylation during zebrafish retinal development is thought to occur by the coupling of APOBEC cytidine deaminase, glycosylase, and GADD45 proteins (Rai et al., 2008; Xiao et al., 2017). A recent study demonstrated that gene knockout of two cytidine deaminases (Apobec2a and Apobec2b) resulted in significantly reduced retinal and optic nerve regeneration. During retinal regeneration, Apobec2a and Apobec2b expression in dedifferentiated Müller glia can induce ASCL1 expression (Powell et al., 2012). This evidence indicates the role of APOBEC cytidine deaminase in retinal and optic nerve regeneration (Powell et al., 2012; Powell et al., 2014). Another study identified dynamic DNA methylation changes during transition from Müller glia to Müller glial progenitor cells (MGPCs) in zebrafish and reported that DNA methylation changes can control the gene expression program of Müller glial reprogramming to be properly activated during retinal regeneration (Powell et al., 2013). This finding indicates that DNA demethylation may be the basis for the reprogramming of Müller glial cells to initiate the regenerative response and may also be the key process to restore the self-healing ability of the mammalian retina (Dvoriantchikova et al., 2019).

The redifferentiation process of Müller glia is affected by DNA demethylation. The promoters of some key genes required for phototransduction are located in highly methylated regions of the Müller glial genome; therefore, transcription of cone photoreceptor–and cone phototransduction–related genes may also require DNA demethylation in the Müller glial genome (Dvoriantchikova et al., 2019). DNA demethylases include the TET family (TET1, TET2, and TET3), which catalyze the sequential conversion of 5-methylcytosine to 5-hydroxymethylcytosine, followed by the conversion of both 5-formyl cytosine and 5-carboxy cytosine to non-methylcytosine (Li et al., 2015), thereby regulating neurogenesis by increasing the expression of target genes (Yao and Jin, 2014). Seritrakul and Gross (2017) reported the impaired differentiation of retinal ganglion cells and photoreceptors in a zebrafish TET2/TET3 double-knockout model, thus revealing the importance of TET2 and TET3 in zebrafish retinal neurogenesis. Moreover, TET3 promoted photoreceptor differentiation in mice (Perera et al., 2015). Therefore, increased TET gene expression during the reprogramming and redifferentiation processes of Müller glial cells may be necessary to promote phototransduction activity and redifferentiation of Müller glial cells into new photoreceptors after retinal injury. Thus, these findings can help us better understand the epigenetic regulatory mechanisms (specifically those related to DNA methylation) during the reprogramming of Müller glial cells.

## 3 Histone modifications in Müller glial reprogramming

Most of the published studies on the retinal regeneration process in zebrafish have focused on the expression of retina-specific genes, which have been identified to be involved in several mechanisms in the reprogramming process, but gene expression is a complex process regulated by multiple factors (Cameron et al., 2005; Qin et al., 2009; McCurley and Callard, 2010). Histone modifications are closely related to the expression of pluripotent transcription factors during the reprogramming of Müller glia (Ueno et al., 2017). Histones are important components of nucleosomes, which are the basic structural unit of chromosomes, and their N-terminal amino acid residues can undergo various covalent modifications such as acetylation, methylation, phosphorylation, ubiquitination, and poly-ADP glycosylation (Strahl and Allis, 2000). Histone modification is involved in the gene regulation of pluripotent factors by affecting the affinity of histones toward the DNA double strands, thereby altering chromatin sparing or the condensed state, or by affecting the affinity of other transcription factors toward structural gene promoters.

The histone deacetylase inhibitor Trichostatin A promoted accessibility of key genetic loci associated with neural development/differentiation in Müller glia and allowed more effective reprogramming (Jorstad et al., 2017). This finding also suggests that histone acetylation can act as a key stimulus switch for the transdifferentiation of Müller glia into retinal neurons. Furthermore, histone acetylation is one of the most important histone modifications that occur mainly at the lysine position at the N-terminal end of histone H3 and H4 (Wang et al., 2009). Additionally, histone acetylation is associated with gene transcription and affects DNA replication and repair. Moreover, histone acetylation is regulated by two classes of mutually antagonistic enzymes: histone acetyltransferases (HATs) and histone deacetylases (HDACs) (Strahl and Allis, 2000). Histone acetylation refers to the fact that HATs neutralize the histone charge by acetylating at histone lysine residues, which weakens the interaction between histones and DNA, allowing the loosening of the chromatin structure and activating gene transcription, whereas HDACs were defined as deacetylate histones, which results in tight binding between histones and negatively charged DNA, dense chromatin packaging, and repression of gene transcription (Chen et al., 2007; Wang et al., 2009). Notably, the activities of HATs and HDAC together determine the level of histone acetylation.

HDACs play an important role in maintaining cellular homeostasis and tissue differentiation. Specifically, HDAC1 plays an important role in embryonic retinal development (Yamaguchi et al., 2005), proper cell division, and maintenance of pluripotency in ES cells (Jamaladdin et al., 2014). However, the mechanism underlying the role of HDAC1 in retinal regeneration remains unexplored. To understand the signaling mechanism of Müller glial reprogramming in the injured mammalian retina, some researchers have analyzed the gene expression of HDACs after retinal injury. The results showed that the HDAC1 protein was present in all retinal cells and confirmed the presence of HDAC1-mediated transcriptional repression of regeneration-associated genes such as ASCL1A and MyCB in these cells (Mitra et al., 2018). HDAC1 expression was significantly decreased after retinal injury, whereas the expression of regeneration-related genes such as ASCL1A and MYC were highly induced through different cellular signaling pathways, which are necessary for the successful dedifferentiation of Müller glia. Interestingly, when HDAC1 was knocked out, the protein levels of regeneration-related genes such as ASCL1A and MYC did not increase but correspondingly rather decreased. Consequently, it led to a remarkable decrease in the number of MGPCs, with deleterious effects on retinal regeneration (Sherpa et al., 2008). This phenomenon is associated with a negative regulatory effect of the delta-notch effector gene HER4.1 on retinal regeneration. HER4.1, in turn, significantly inhibits LIN28A. LIN28A is an important pluripotency-inducing factor. A decrease in its expression level leads to an increase in LET-7 microRNA, which blocks the translation of ASCL1a and MYC (Ramachandran et al., 2010; Kaur et al., 2018), finally inhibiting the degree of dedifferentiation and the level of proliferation in Müller glia. Additionally, HDAC1 mRNA levels recovered around the fourth day after retinal injury in the vicinity of the injury site (Mitra et al., 2018), which has a repressive effect on the subsequent transcription of regeneration-related genes. Furthermore, HDAC1 expression decreases in the early stage after retinal injury, which promotes the dedifferentiation of Müller glia into MGPCs by upregulating the expression of several regeneration-related genes and cytokines, whereas HDAC1 expression increases in the late stage after retinal injury, which inhibits the degree of proliferation of MGPCs. Hence, HDACs integrally regulate retinal regeneration.

In addition, the reprogramming process is also influenced by the microenvironment. Glucose and oxygen are important regulators of stem cell fate, and somatic reprogramming and pluripotency undergo transition from oxidative metabolism to anaerobic glycolysis (Folmes et al., 2011). While NAD+-dependent histone deacetylase SIRT6 is a key regulator of glucose homeostasis, its deletion or inactivation shifts toward anaerobic glycolysis by upregulating the expression levels of the glucose transporter protein GLUT1 and several glycolytic genes (Zhong et al., 2010). Furthermore, SIRT6 regulates the expression of several core pluripotency genes through the deacetylation of ACH3K56 and ACH3K9 during ES cell differentiation (Etchegaray et al., 2015); furthermore, SIRT6 plays a key role as a regulator of these cellular pluripotency genes. The high-mobility group box 9 transcription factor (SOX9) is essential in the establishment and maintenance of neural stem cells in the embryonic and adult central nervous system (Scott et al., 2010). It plays a crucial role in triggering the transition from neurogenic to glial-derived programs in the germinal zone of different neural tissues. During retinal formation, SOX9 is expressed in pluripotent progenitor cells, but it is expressed only in Müller glia and RPE cells in the adult retina. Thus, high glucose induced a decrease in SIRT6 expression levels and an increase in H3K9 acetylation levels and pluripotency factor SOX9 levels (Zorrilla-Zubilete et al., 2018). Moreover, inhibition of SIRT6 expression with siRNA increases SOX9 expression (Sanhueza Salas et al., 2021), thereby promoting Müller glial reprogramming. The above evidence suggests that the histone deacetylase SIRT6 can regulate the retinal regeneration process after retinopathy by regulating the expression of regeneration-associated neurotransmitters in Müller glia.

The key gene regulatory networks of retinal regeneration can be unraveled with temporal and spatial precision only by gaining a better understanding of the activity and mechanisms of action of epigenomic modifiers such as HDACs and their associated genes during retinal regeneration. The cellular and molecular regulatory mechanisms of zebrafish retinal regeneration could lay the groundwork for the treatment of mammalian retinal damage to facilitate the development and application of mammalian retinal regeneration therapy. Moreover, different studies have identified trimethylation of H3K27 (Mitra et al., 2018) and phosphorylation of H3S10 and H3S28 (Sawicka and Seiser, 2012), both of which are associated with Müller glial cell reprogramming, which further suggests that histone modifications play an important role in regulating Müller glial cell reprogramming.

## 4 Non-coding RNA in Müller glial reprogramming

Non-coding RNA (ncRNA) refers to RNA that does not encode a protein, but this does not mean that such RNA does not contain information or has no function. ncRNA includes ribosomal RNA, transfer RNA, small nuclear RNA, small nucleolar RNA, and microRNA (miRNA) with known functions, and it may also include RNA with unknown functions (Mattick and Makunin, 2006; Esteller, 2011; Li et al., 2013; Li et al., 2020). To this end, a significant number of investigations conducted during the past decade have transformed our perception of ncRNAs from “junk” transcription products to functional regulatory molecules that regulate cellular processes including chromatin remodeling, transcription, post-transcriptional modification, and signal transduction. Furthermore, they are involved in gene regulation at different levels, ranging from epigenetic silencing to post-transcriptional regulation of mRNA stability. Thus, ncRNAs are the key regulators of various physiological and pathological programs (Guttman and Rinn, 2012).

miRNAs are a class of conserved ncRNAs that regulate post-transcriptional gene silencing by targeting the 3′-untranslated region of mRNAs and are essential for almost all biological processes (Friedman et al., 2009; Ha and Kim, 2014). As with other tissues, miRNAs play an important role in retinal regeneration. Using a zebrafish model with Dicer knockdown and light-induced damage, Rajaram et al. (2014) demonstrated that the dicer-dependent miRNA biogenesis pathway is essential for normal retinal regeneration in zebrafish. In this regard, by examining the loss of regenerative function in the regenerating retina, the authors elucidated the role of differentially expressed miRNAs in regulating the number of proliferating Müller glia–derived neuronal progenitors during retinal regeneration in adult zebrafish. In this model, morpholino-mediated knockdown of upregulated miR-142b, miR-146a, miR-7a, miR-27c, and miR-31 reduced the number of neuronal progenitors proliferating in zebrafish retina upon light irradiation. This evidence indicates that these miRNAs may promote the progression of Müller glial dedifferentiation–mediated retinal regeneration. Recently, additional studies have demonstrated that overexpression of miR-25 (Wohl et al., 2019; Liu et al., 2021) and miR-124-9-9* (Wohl and Reh, 2016b) enhances ASCL1-induced reprogramming in cultured Müller glia.

Rajaram et al. (2014) highlighted the decreased expression levels of 23 miRNAs during the proliferation period, including miR-23a, miR-143, and miR-145. We speculate that the downregulated miRNAs may act as guardians of cell identity and play a role in preventing cell dedifferentiation. Interestingly, miR-23a is highly expressed in Müller glia freshly isolated from PN11-12 mice (Wohl and Reh, 2016a). Although miR-145 is enriched in murine cultured Müller glia (PN8), this miRNA is downregulated in different models of Müller glia dedifferentiation (Katsman et al., 2012; Rajaram et al., 2014; Quintero et al., 2016). Another experiment demonstrated that Oct4 can participate in the reprogramming of Müller glia by inhibiting miR-143/145 (Sharma et al., 2019). Zhao et al. (2017) and Tao et al. (2016) reported that upregulated expression of Lin28b could promote reprogramming of Müller cells to retinal progenitor features by causing downregulation of LET-7 miRNA. Wohl et al. (2019) conducted an *in vivo* mouse study and reported that antagonizing LET-7 induced sc11 expression and that 40% of mature Müller glia were converted to a neuronal/RPC phenotype. These results indicate that the differential expression of miRNAs between Müller glia and RPC leads to differences in their neurogenic potential, and manipulation of miRNAs provides a novel tool to reprogram Müller glia to promote retinal regeneration. Further reports confirmed that LET-7 regulates the translation of shha, shhb, Smo, Ptch1, and zic2b mRNAs in the damaged zebrafish retina. The Shh signaling pathway is required for the induction of pro-regenerative gene expression cascades, including ASCL1a, Lin28a, MMP9, Foxn4, and zic2b, during retinal regeneration (Kaur et al., 2018). Later, Kara et al. (2019) demonstrated that the miR-216a-Dot1l regulatory axis negatively regulates glial cell reprogramming during Müller glial retinal regeneration through the classical Wnt signaling pathway. In a rat model of optic nerve crush (ONC) injury, Li et al. (2019) revealed that miR-21 inhibition could promote the recovery of retinal ganglion cell function by regulating Müller glial cell proliferation after ONC injury. It is thus evident that downregulation of certain miRNAs can provide a proven strategy for regulating the regenerative process of glial reprogramming in Müller cells.

The miR-29 family and miR-200 family appear to be consistently associated with Müller glia (Chung et al., 2016; Quintero et al., 2016; Zeng et al., 2017; Sharma et al., 2019). miR-29a regulates the proliferation and differentiation of RPCs by targeting Rbm8a (Zhang et al., 2017), whereas miR-29b may be involved in regulating apoptosis in Müller glia (Zeng et al., 2017), and miR-29c contributes to gliogenesis by silencing transcriptional repressors (REST) (Xia et al., 2019). miR-200b can mediate Oxr1 downregulation leading to Müller glial cell death (Murray et al., 2013). Conversely, blocking transforming growth factor-β signaling elevates the level of miR-200a/b mediating the reprogramming of Müller glia (Sharma et al., 2020). These miRNAs appear to be promising candidates for exploring miRNA-based regulation of reprogramming in Müller glia.

## 5 Discussion

Some studies have reported retinal injury–induced reprogramming of Müller glial cells, wherein the process involves multiple epigenetic modifications. These epigenetic modifications control multiple signal transduction pathways and gene regulatory networks. The response of Müller glial cells to retinal injury is a complex and highly regulated multistep process, each of which requires the expression of specific genetic programs (Gorsuch et al., 2017; Hoang et al., 2020).

The epigenetic plasticity of Müller glia in zebrafish enables the glia to undergo a reprogramming process in response to injury and produce RPCs, which later differentiate into all types of retinal cells to repair the damaged retina and restore vision. However, because of the limited epigenetic plasticity, the reprogramming ability of mammalian Müller glial cells is weak (Bonilla-Pons et al., 2022). Epigenetic regulation of reprogramming in mammalian Müller glial cells produced a small number of retinal neurons, bipolar cells, and rod photoreceptors in late stage of growth (VandenBosch et al., 2020; Sanhueza Salas et al., 2021). Therefore, epigenetic modifications in the reprogramming of Müller glial cells are the focus of current research. Multiple molecular and signaling pathways are involved in Müller glial reprogramming and affect retinal physiological and pathological processes. Therefore, we summarize the regulatory network of DNA methylation, histone modifications, and microRNAs participating in Müller glial reprogramming to mediate retinal regeneration, which is necessary as an option for the prevention and treatment of retinal diseases. In most cases, studies have revealed the role of epigenetic modifications through spontaneous reprogramming of zebrafish Müller glia and induction of *in vitro* reprogramming of mouse Müller glia, while many limitations remain in our understanding of the molecular mechanisms underlying the reprogramming of mammalian Müller glia into stem cells. Müller glia reprogramming differs significantly in different species (Hoang et al., 2020). The study of the exact differences in these mechanisms between different species is another important area to correlate the results obtained in different animal models with those obtained in humans. Recent studies have suggested that retinal damage activates common and distinct genetic programs in zebrafish and mouse Müller glia. Mining these data will undoubtedly uncover important new avenues of research that will help unravel the underlying mechanisms of Müller glial programming and thus advance the goal of repairing the human retina through intrinsic cellular sources.

**FIGURE 1:**
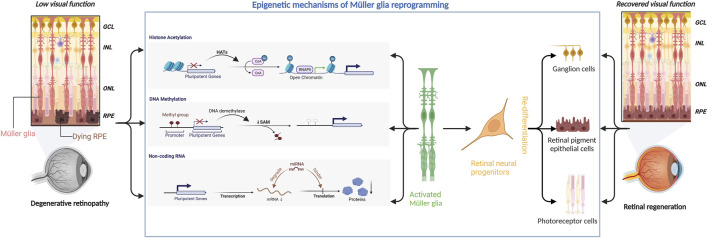
Epigenetic mechanisms underlying Müller glial reprogramming. Retinal degenerative diseases can severely compromise the visual function of the affected patients. Epigenetic mechanisms (including histone acetylation, DNA methylation, and microRNA) can regulate the expression of pluripotent genes to activate Müller glia to initiate the reprogramming process. Müller glia then differentiates into retinal neural progenitor cells, which subsequently migrate to the damaged area and redifferentiate into retinal ganglion cells, retinal pigment epithelial cells, and photoreceptors, thereby aiding in retinal regeneration and restoration of visual function.

In conclusion, Müller glial reprogramming is a complex process involving multiple participants that may vary among different retinal diseases. There is no single factor that can effectively promote the regeneration of damaged neurons in retinopathy. Epigenetic modifications affect multiple targets and are effective tools for Müller glial reprogramming to promote retinal regeneration (Figure 1).
